# Determinants of Farmland Abandonment on the Urban–Rural Fringe

**DOI:** 10.1007/s00267-020-01258-9

**Published:** 2020-02-08

**Authors:** Ting Zhou, Eric Koomen, Xinli Ke

**Affiliations:** 1 Department of Spatial Economics, Vrije Universiteit Amsterdam, De Boelelaan 1105, 1081 HV Amsterdam, The Netherlands; 2 Department of Public Administration, Huazhong Agricultural University, No. 1, Shizishan Street, Hongshan District, Wuhan, 430070 PR China

**Keywords:** Farmland abandonment, Peri-urban agriculture, Urbanization, Urban proximity, Wuhan, China

## Abstract

China’s urban explosion has resulted in a substantial loss of agricultural production on the fringes of many cities. Farmland is not only converted into urban uses but also taken out of production because it has lost its value for those who can farm it. This farmland abandonment process has received little research attention. This paper studies the abandonment of farmland around the rapidly urbanizing city of Wuhan and aims to identify its important determinants based on an extensive field survey among local farmers. Around 800 semi-structured field interviews were conducted to capture the parcel and location characteristics, farming practices, and household characteristics. Important parcel-related drivers of land abandonment are lack of family members to work the land and fragmentation of parcels. Spatial characteristics are less important, except for the presence of certain soil types that favour cultivation and designation under the farmland protection policy. The planted crop species and the option to transfer land to other farmers are important farming practices to continue cultivation. Moreover, farmers with a higher farming income and lower education levels are less likely to abandon their farmland. We suggest that land use policies can help in preventing further farmland abandonment by steering urban development away from the most suitable soils for farming and concentrating development to limit the fragmentation of parcels. Strengthening the land market and removing the remaining barriers for farmers to transfer land to colleagues can further help to keep farmland in production.

## Introduction

Farmland abandonment is taking place in many countries around the world and has received substantial attention from researchers and policy makers because of its environmental and socio-economic impacts (e.g. Rey-Benayas et al. 2007; Van der Zanden et al. 2016). This process can have positive consequences for the supply of ecological services, for example, soil recovery (Benayas et al. 2007) and water retention (Sileika et al. 2006). On the other hand, farmland abandonment can also result in the deterioration of agro-ecosystems and loss of biodiversity in agricultural landscapes (Plieninger et al. 2016; Stoate et al. 2009), threaten food security (Meyfroidt et al. 2016), lead to regional economic decline (Gellrich et al. 2007; Renwick et al. 2013), and widen the urban–rural income gap (Müller and Munroe 2008).

Farmland abandonment is usually associated with marginal areas where farming costs are no longer compensated by the profits from production (Díaz et al. 2011; Gellrich et al. 2007). These marginal areas are characterized by environmental, economic, and social constraints (MacDonald et al. 2000; Renwick et al. 2013; Rey-Benayas et al. 2007; Strijker 2005; Van Vliet et al. 2015). The key determinants of farmland abandonment in such areas are: (1) undesirable physical and climatic characteristics, such as poor soil quality, steep slopes, high elevation, and limited precipitation (Díaz et al. 2011; Gellrich et al. 2007; Osawa et al. 2016); (2) unfavourable socio-economic conditions, which include demographic change (e.g. an aging population) and poor accessibility (Anderson 1992; Prishchepov et al. 2013); and (3) specific farmer characteristics, such as age, education level, and occupation (Bartolini and Viaggi 2013; Lieskovský et al. 2015). Obviously, the exact combination of determinants may vary across time and space (Rey-Benayas et al. 2007). In most of the reviewed literature, farmers have been portrayed as rational agents who strive to maximize their income or minimize their loss (Díaz et al. 2011; Gellrich et al. 2007). This implies that the cultivation of farmland only ceases when the agricultural profits decline to the zero-profit level or when a higher income can be obtained elsewhere (Gellrich et al. 2007; Kristensen et al. 2004). In fact, farmers reportedly persist in traditional cultivation practices until the returns are negative and/or the cultivation costs result in substantial financial losses (Strijker 2005).

While low-intensity farming and land abandonment are often linked to marginal conditions of land in remote locations, these phenomena are also found on the urban fringe in urbanizing and industrializing regions, where the farming conditions are not necessarily marginal and certainly do not suffer from being remote. Sinclair (1967) was the first to develop an explanatory model for lower land use intensity near cities, countering the classic theories of Alonso (1964) and Von Thünen (1966) that posited land use intensities to be highest near cities (marketplaces) and to decline with increasing distance from them under transport cost constraints. A possible explanation for the low-intensive use of agricultural land on the urban–rural fringe is that farmland adjacent to urban areas has become too expensive for agricultural purposes. Farmland prices near cities are rising due to the increasing demand for land for urban development (Cavailhès and Wavresky 2003; Chicoine 1981; Sinclair 1967). In addition, peri-urban farmland owners may wait for the opportunity to sell their land or speculate on rising land prices and prefer temporary farming practices with less investment until the moment when they can sell for their preferred price (Sinclair 1967). Alternatively, small parcel sizes, inconvenient parcel shapes, and fragmentation of parcels (Heimlich and Anderson 2001; Sklenicka et al. 2014) or the advent of recreational farmers have been suggested to explain low farming intensities (Heimlich and Barnard 1992). Interestingly, this small-scale type of farming is increasingly appreciated around cities, as it provides nearby citizens with the opportunity to experience the cultivation and harvesting of crops, see small farm animals, or gain agricultural knowledge (Zasada 2011). This new rise in urban agriculture may keep agricultural land in peri-urban areas in production (Cavallo et al. 2016; Dieleman 2015), but it is unclear whether the income generated by these activities will be able to help farmers to pay the higher land rents near cities in the long run.

This paper aims to explain farmland abandonment on the urban–rural fringe in China. There exists considerable evidence that farmland around major Chinese cities is relatively fertile (due to past investments) and used more intensively; see e.g. Deng et al. (2006) and Tan et al. (2007). However, evidence has been found recently for a decline in agricultural land use intensity and the abandonment of farmland around Chinese cities (Jiang et al. 2013). This is not only influenced by farmers’ expectation of land uptake for urban development in near future but also as a result of more indirect changes in farmers’ behaviour through, for example, the pursuit of alternative income-generating activities and lower investments in capital and effort for farming practices. A better understanding of the underlying determinants of farmland abandonment on the urban–rural fringe may provide policy makers with guidance for developing policies that support the sustainable use of farmland around cities.

Understanding the drivers of peri-urban agricultural land abandonment in China is particularly interesting as most studies on declining agricultural intensities and farmland abandonment on the urban–rural fringe have been performed in Europe and the United States. So it is unclear whether the explanations proposed for these cases are applicable to the economic, spatial, social, and institutional contexts of China. One of the most notable differences is that land on the urban fringe in China belongs to rural collectives and is managed by households that have a contract to use specific plots. Ding (2003, 2007) and Wang et al. (2015) provided an extensive discussion on the recent changes in these land use rights and the functioning of the Chinese land market. This government-controlled market does not necessarily result in the higher land rents on the urban–rural fringe that are found to hamper agricultural production opportunities in, for example, Europe and the United States (Cavailhès and Wavresky 2003; Chicoine 1981; Sinclair 1967). Nevertheless, the hybrid Chinese land market that combines government controls with market-based transfers also favours urban conversion over agricultural use and has been suggested to result in over-conversion of farmland (Tan et al. 2011). Even compensations would be paid to farmers for the lost farmlands, they were quite low compared with their market values if the farmlands were converted to non-agricultural uses (Ho and Lin 2003; Lichtenberg and Ding 2008). To a certain extent, the post-socialist European countries may offer an analogy for the Chinese context. From earlier research on land use intensity in socialist cities with a planned economy, we know that urban population densities could differ strongly from the classic Alonso-style gradients and exhibit higher densities further away from the city (e.g. Bertaud and Renaud 1997). Moreover, little economic incentive existed to develop excess land or unused parcels within cities (Dale-Johnson and Brzeski 2001), indicating that land was not used in the most efficient way. With the evolution of a land market after the transformation to a market-based economy, the prices of land transactions began to rise and started to differentiate between different types of use (e.g. for commercial, industrial, and agricultural transactions), while the gradients describing price as a function of the distance to the city centre began to steepen (Dale-Johnson and Brzeski 2001). For China this suggests that the recent land market reforms may also result in higher prices for land transactions around cities. The strict separation of land ownership (which belongs to collective organizations of farmers) and land use rights of individual farmers (see Tan et al. 2005) implies, however, that individual farmers will not profit from selling their land. Nonetheless, the possibility that their land may be converted into urban use in the near future may influence their willingness to invest time and resources in farming practices.

In this study we focus on farming households on the urban–rural fringe of the rapidly urbanizing city of Wuhan and analysed the factors that steer their decision to take land out of production. From the above we hypothesised that parcel and location characteristics describing farmland conditions will be important determinants. In addition we pay ample attention to the role of farming practices and household characteristics, including the importance of off‐farm income (from, for example, employment in the city), in their decision to leave part of their land uncultivated (Lichtenberg and Ding 2008).

## Method

### Study Area

The city of Wuhan in Central China is the case study area for this paper. Wuhan has around 10 million inhabitants and is characteristic of the most recent wave of rapid urbanization in the country. It experienced annual population growth of 1.4% from 2010 to 2014, which is similar to the average growth rate of 1.2% of the 21 Chinese municipalities with the largest built-up area (National Bureau of Statistics of China 2016).

Wuhan is the capital city of Hubei province and one of the ten mega cities in China with over 10 million inhabitants. More than 80% of its residents live in the urban area (National Bureau of Statistics of China 2016). According to the local land use plan, more than one-tenth of the farmland should be developed into highly productive fields by 2020, and four times more is expected to be developed in the longer term (Wuhan Natural Resources and Planning Bureau 2011). Moreover, Wuhan is planning to develop a modern urban agricultural system based on multiple uses of farmland by, for example, combining food production, recreational horticulture, and eco-tourism (Wuhan Natural Resources and Planning Bureau 2011).

At the same time, urban development is expected to increase in the near future (Hoornweg 2013). This process is associated with increasing rates of migration from agricultural to urban areas following a rise in the opportunity cost of farming. This results in the conversion of farmland into urban uses but may also threaten the sustainability of agricultural landscapes and jeopardize the agricultural development objectives of the region (Wuhan Natural Resources and Planning Bureau 2011).

**Fig. 1:**
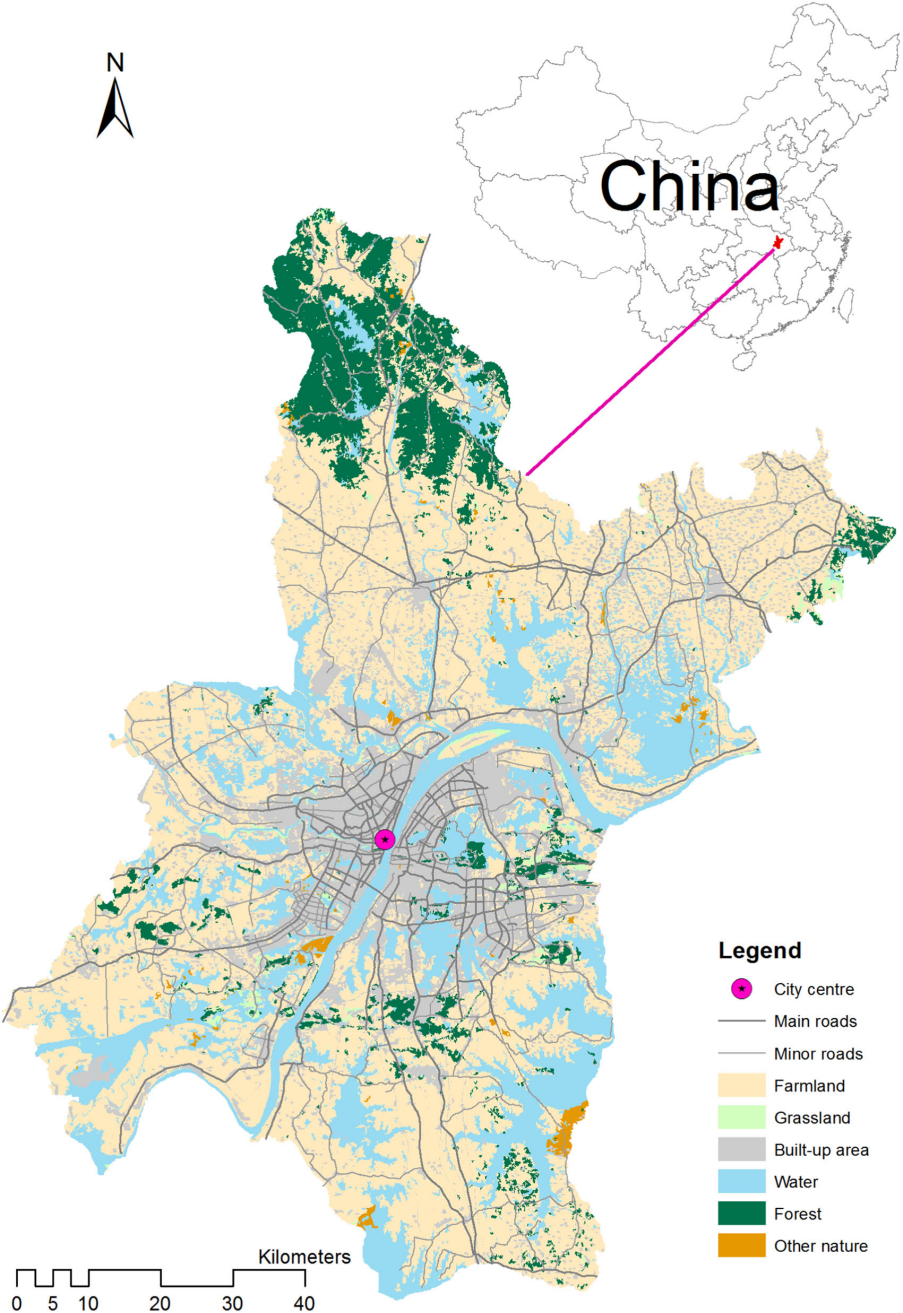
Land use in Wuhan municipality

The municipality of Wuhan covers an area of 8569 km^2^, half of which is currently in agricultural use (Hubei Provincial Bureau of Statistics 2016) (Fig. 1). The city is located at the eastern extremity of Jianghan Plain, with an elevation ranging between 7 and 61 m. The climatic conditions are favourable for agriculture: temperatures are fairly mild (the mean annual temperature is around 17 °C), with sufficient sunshine (1865 h on average per year) and rainfall (1315 mm average per year) for year-round production (National Meteorological Information Center 2015). The city lies at the confluence of the Yangtze and Han rivers, which have deposited fertile soils classified as paddy soil, yellow brown earth, swampy soil, and fluvo-aquic soil. Especially the latter type is considered to be favourable for the production of maize, wheat, and cotton (Qin et al. 1998). The main agricultural products in the region are rice, wheat, cotton, corn, sweet potatoes, peanuts, and oilseed rape (Hubei Provincial Government 2015). The agricultural production from local farmers within the Wuhan municipality in terms of value sold has risen in recent years (Hubei Provincial Government 2015).

Several wholesale markets are located in the city of Wuhan, where farmers can sell their produce alongside food products imported from other regions. The total value of the agricultural products sold at these markets is many times higher than the value of agricultural production within the municipality (Hubei Provincial Bureau of Statistics 2016), indicating the importance of Wuhan as a distribution centre for agricultural production.

### Study Design and Data Collection

To study the factors that contribute to farmland abandonment, we set up an extensive field survey among local farmers. Around 800 semi-structured field interviews were held in 2015 with farming households along the urban–rural fringe to capture the farming practices, the characteristics of their parcels, and the factors affecting their land management decisions. Subsequently, regression analysis was conducted to link the share of abandoned farmland area per household with the financial and social characteristics of the households and the spatial conditions of the farmland. In this study farmland was defined as abandoned if agricultural practices have ceased completely and there has been no financial, labour, or other input used for agricultural production during the past 12 months. Farmland with trees was considered to be abandoned if trees or their products have not been used for commercial purposes and the land has not required agricultural input, such as labour.

The urban–rural fringe that we considered in this study is approximately a 10 km wide zone that connects the contiguous and predominantly urban area of Wuhan city with its mostly agricultural surroundings. Farms were visited along transects of around 10 km length fanning out in different directions that run perpendicular to the border of the urban core. As land use practices and socio-economic characteristics of farmer households were found to similar in the same village, only two farmer households were selected randomly for interviews in one village. If the selected household was not available, another household next door was interviewed. At each selected farm residence, we initially introduced our study purpose and held an oral interview of around 15 min with one of the family members, in which we posed a set of predefined questions. The respondent was either the head of the household or—if unavailable—the person most familiar with the farmland conditions. From the ~1000 farms selected along the selected transects, around 200 farming households had recently converted their farmland into other uses, such as road infrastructure or residences. These households were excluded from the survey, but almost all other farm households along the transects participated in the survey. From the 797 responses, 592 (75%) were found to be complete and valid for further analysis.

The questionnaire was set up to incorporate the most important drivers of agricultural land abandonment on the urban–rural fringe documented in literature. Foremost, these relate to the size, number, and location characteristics of the farmers’ parcels, as smaller, scattered, and more fragmented plots of land—which are the likely result of the ongoing urbanization processes—may negatively influence the farming potential (Sklenicka et al. 2014).

A second set of driving forces relates to the location of the farm. Being close to the city centre, for example, may have negative impacts, such as pollution, congestion, theft, vandalism, high land prices, and uncertainty about future developments (as described by, amongst others, Eagle 2009; Heimlich and Anderson 2001; Sinclair 1967). On the other hand, the proximity to the food markets located in the city can benefit farmers. We therefore include the distance to the border of the main urban districts in our analysis. Proximity to a road network can also be beneficial in providing easy access to markets and suppliers (Diogo et al. 2015) and was included in this study as the distance to minor roads rather than to the much sparser major roads (Díaz et al. 2011). In addition, the water supply is considered to be important for cultivation (for example for vegetables; see Terres and Nisini 2013), and we tested its impact on abandonment by including the distance to the nearest water body in our analysis. To account for the possible impact of the physical and climatic characteristics of farm locations, we also included references to the soil type, water availability (being a flooded paddy field as opposed to being dry land), and slope. We acknowledge, however, that these are less likely determinants of abandonment in our case, with fairly homogeneous and generally favourable conditions, than in the wealth of literature that has described abandonment in more marginal and remote areas (Gellrich et al. 2007; Rey-Benayas et al. 2007). As we assume farms on the urban fringe to be prone to being converted into other types of use, we asked the farmers whether their farmland belongs to a preserved farmland zone or not.

In relation to farming practices, we asked the farmers about the crops that they produce, the area left uncultivated for the past 12 months, the input used for farming, including the number of people working on the farm, and the area of land that has been transferred out (leased to other farmers). The four main crop types that we distinguish are staple food, cash crops, vegetables and fruit, and ornamental trees. To raise the farming profits, there are incentives for farmers to transform traditional farming systems (producing staple food) into more diverse agricultural systems that provide high-value products, such as vegetables and fruit (Van den Berg et al. 2007). Moreover, we investigate whether the diversity of crop species helps to mitigate farmland abandonment. Crop diversity is a known strategy to minimize risk (Di Falco and Perrings 2005) and maintain agricultural production by coping more effectively with insects, diseases, and environmental stress (Benin et al. 2004; FAO 2014; Salvatore and Charles 2005). A Shannon index is calculated to describe crop diversity (Mahy et al. 2015). Following several land policy reforms, the option for farmers to transfer out part of their contracted land has become increasingly popular in recent years (Wang et al. 2015). About half of the transfers are arranged with family members or relatives and do not necessarily involve substantial rent payment (Wang et al. 2015). However, these reforms are believed to have established a form of land market that is expected to transfer land from low-productivity farming households to high-productivity ones (Deininger and Jin 2005) and thus to limit the need for land reallocation (Zhang et al. 2011). While farmers in some regions in China benefit from agricultural subsidies (see, for example, Frederick et al. 2005; Zhang et al. 2018), the respondents in our survey indicated that they considered these to be very low and not influencing their farming practices. In addition, agricultural subsidies mainly go to grain producers in China, with agricultural input subsidies constituting the largest share (Gale 2013; Huang and Yang 2017). Hence, agricultural subsidies are likely to affect only the grain producers in the survey region. We therefore excluded this aspect from further analysis.

In addition, questions were asked to gain a better understanding of the socio-economic situation of the households. These questions relate to their income, education level, and embedding in the local community. A higher income generated by farming activities is assumed to provide an incentive to continue farming. Income from off-farm sources can result in agricultural land abandonment (as documented for Puerto Rico by Rudel et al. 2000), but this relation is not always straightforward. In many developed countries, large shares of off-farm activities coincide with continued farming activities: 90% of the average farm household income originated from off-farm sources in the United States in 1999 according to Heimlich and Anderson (2001), while 45% of Irish farms were sustained by incomes generated outside agriculture according to Kinsella et al. (2000). Individuals’ education level is expected to capture part of their socio-economic status and potential for accessing better-paid off-farm jobs. Their embedding in the local community is proxied by asking them to estimate the number of persons in their local community with whom they interact in social activities, such as the sharing of food and agricultural knowledge or the provision of unpaid labour. This indicator follows the approach of Lovell (2010), which was developed to describe the social function of farmland, in which we assume that those with a more extensive network in the local community are more likely to continue farming.

An initial version of the questionnaire was pretested with a small number of farmers on the urban–rural fringe of Wuhan in early November 2015. Based on their comments, some questions were clarified and adapted to the specific local conditions, for example by including a reference to having multiple parcels. The survey was conducted in late November and December 2015, when farming activities did not demand too much time from the respondents. See Appendix 1 for a transcription of the full questionnaire that was used in the survey.

**Fig. 2:**
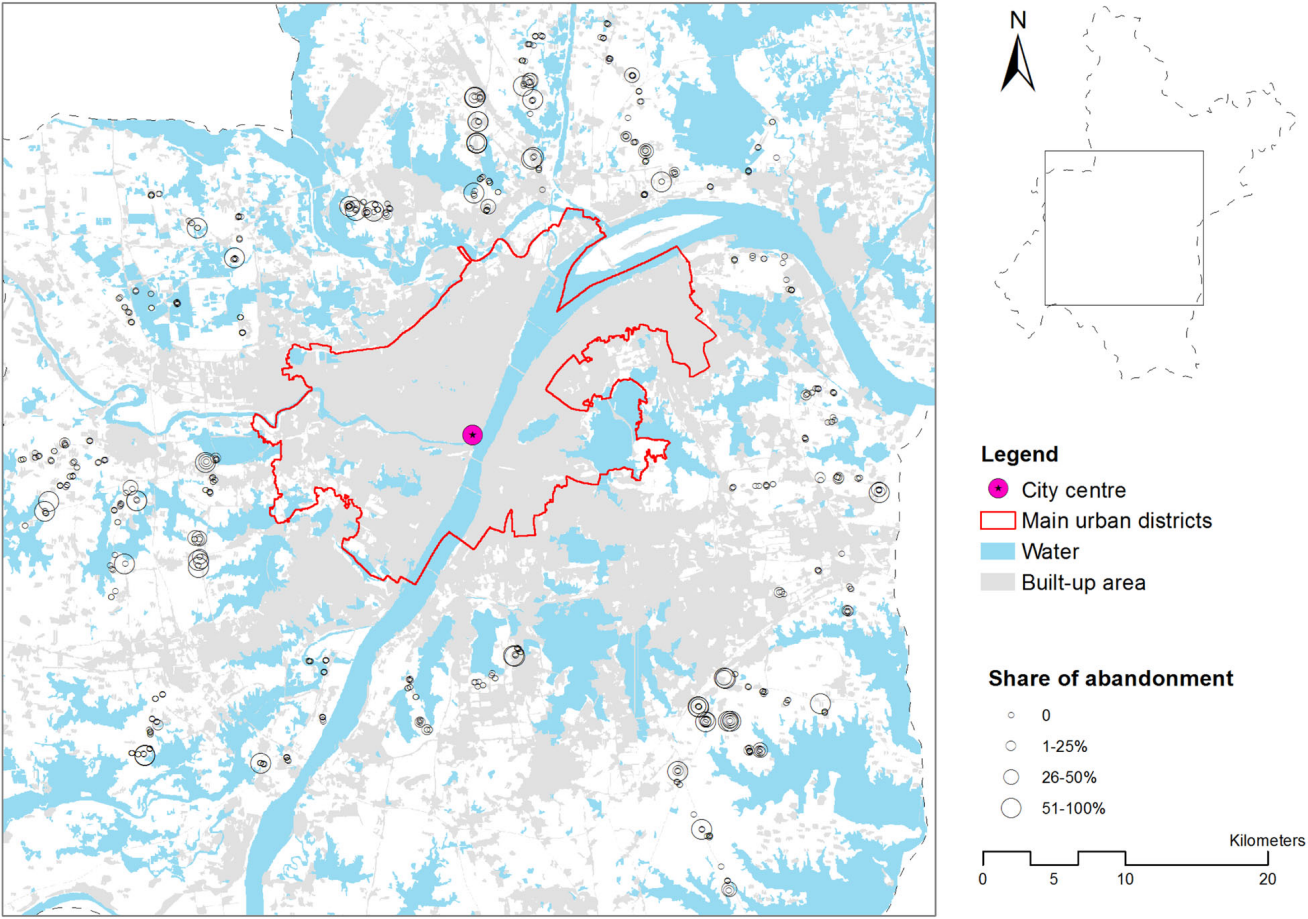
Location of the farming households included in the analysis and their shares of abandoned farmland. This representation does not indicate the size of the farm or the number of parcels that are managed

For each household the geographic coordinates were recorded with a mobile GIS application (ViewRanger) to enable us later to enrich the information from the survey with spatial data sets that describe the local soil condition, slope, and accessibility characteristics at the farm residence location. Figure 2 shows the location of the farming households included in our analysis and their share of abandoned farmland. The sampled locations are distributed fairly evenly around the urban–rural fringe of Wuhan and provide sufficient spatial variation across the study area.

### Data Analysis

#### Spatial Analysis

A standard desktop geographical information system (ArcGIS 10.4) was used to map and analyse the spatial conditions at the farm household locations. The following data sources were used to characterize the local farming conditions: (1) the soil type from the China Soil Database (gis.soil.csdb.cn); (2) the slope from the Shuttle Radar Topography Mission (SRTM)—a joint mission conducted by NASA (the National Aeronautics and Space Administration of USA) and the NGA (the USA National Geospatial-Intelligence Agency); (3) the land use from the Data Centre of Resources and Environment, Chinese Academy of Science (Liu et al. 2010); and (4) the minor road network from the Traffic Atlas of Wuhan. To make all the data sets consistent in format and spatial resolution, they were converted into a 100 × 100 m raster to match the land use data.

The spatial data sources were used to describe the physical conditions at the farm location and calculate the distance-related variables using a Euclidean distance function. To calculate the distance to the urban core of Wuhan, we selected six main urban districts (from the city’s thirteen municipal districts), which were located in and around the city centre and had the largest built-up area fraction. In addition, the distance from the farmhouse to the nearest water body was calculated.

#### Regression Analysis

To link farmland abandonment and the observed levels of farming intensity with the household characteristics and the spatial conditions of the farmland that they use (e.g. the parcel size and distance to the city), we applied the ordinary least squares regression (OLS Regression). As we are interested in the interplay of spatial and household conditions, we performed our analysis at the household level. Consequently, we do not explain the impact of determinants on the abandonment of individual parcels but rather the share of abandoned parcels per households. We applied OLS regression to analyse the marginal effects of various determinants on the share of abandoned parcels per household.

## Results

Table 1 provides a summary of the variables that were included in this study. It indicates that farmland abandonment is fairly widespread: on average 9% of the farmland area of each household is abandoned. The parcel characteristics at the farms differ considerably: on average farms have around 0.4 ha of each parcel, but the largest farm is 3.9 ha. While the sampled farms on average have five parcels, this ranges up to 32 separate plots of land. This variation is also apparent from the largest patch index, which captures fragmentation: while the largest parcel in some cases only measures 4% of the total cropland area, on average this is 52%. Fragmentation is furthermore reflected in the distance to the furthest parcel that is low on average (350 m) but ranges up to 15 km.

**Table 1:** Descriptive statistics of the farming households, their farmland, and the abandonment determinants

Determinant	Mean	St. dev.	Min.	Max.
Dependent variable				
Share of abandoned area per household	0.09	0.23	0	0.99
Parcel characteristics				
Total farmland area per household (ha)	5.43	5.91	0.02	58
Number of parcels per household	5.26	5.21	1	32
Farmland area per person (ln (ha))	**−**0.55	1.89	−4.79	3.14
Largest parcel index (fraction)	0.53	0.32	0.04	1
Distance from the farmhouse to the furthest parcel (ln (m))	5.86	1.65	1.61	9.62
Location characteristics				
Distance from the farmhouse to the main urban districts (ln (m))	9.14	0.53	7.66	10.25
Distance from the farmhouse to the nearest minor road (ln (m))	3.57	2.69	1.61	8.62
Distance from the farmhouse to the nearest water body (ln (m))	5.92	1.22	1.61	7.57
Fluvo-aquic soil at the household location (1, 0)	0.33	0.47	0	1
Paddy field (1, 0)	0.58	0.49	0	1
Slope at the farmhouse location (degree)	2.09	1.31	0	9.65
Located within a farmland preservation area (1, 0)	0.11	0.31	0	1
Farming practices				
Staple food dominated (1, 0)	0.23	0.42	0	1
Cash crop dominated (1, 0)	0.14	0.34	0	1
Vegetable and fruit dominated (1, 0)	0.37	0.48	0	1
Ornamental tree dominated (1, 0)	0.15	0.35	0	1
Mixed crop species	0.11	0.32	0	1.22
Area transferred out (ln (ha))	0.18	0.63	−2.30	4.01
Household characteristics				
Household size (persons)	4.51	2.41	1	19
On-farm income per person (ln (yuan))	3.83	3.58	0	10.71
Adult family members with at least high school education (fraction)	0.20	0.25	0	1
Number of community connections (ln)	4.60	1.21	0	7.31

Observations = 592

To characterize the location of the farm, we calculated three distance-related variables that express proximity to the main urban districts of Wuhan, the road infrastructure, and the nearest water body. The distance to the main urban districts shows an average of 3 km, with some farms located directly at the city edge and others located in more rural areas 9 km from the main urban districts. The distance to the nearest minor road shows a fairly short range, from being right next to the roadside to a maximum distance of around 6 km. Water is even more omnipresent, with an average distance to the nearest water body of only 0.5 km and a maximum distance of just 2 km. To suppress this variation, we applied a logistic transformation. This emphasized the variation at the low end of the distribution and assumed that the difference between long and very long distances is less relevant. Alternative specifications of our statistical model confirmed this assumption. In addition, we included two geographical variables that capture the local farming conditions: the presence of favourable fluvo-aquic soils and the slope. The latter indicated that the area is mostly flat and only in some cases the slopes reach a still fairly gentle maximum of around 10°.

Concerning the farming practices, we found that only 14% of the surveyed farms had transferred out part of their land. This is only half of the national average of around 30% in 2015 according to Wang et al. (2015). The transferred-out area per farm ranges from 0.01 to 6.93 ha but is fairly small in the majority of cases (only in <2% of the farms is this area >10 ha).

There is a big gap in on-farm income between the surveyed farmers: almost half reported that they had acquired no income at all, while most of the remaining households had earned <5000 yuan/person. Only the top 4% had an income between 10,000 and 45,000 yuan/person. On average, one out of five adult household members had completed at least high school. Most farming households have an extensive social network: half of them are in touch with around 100 persons in their neighbourhood, and about 10% of them have a social network that consists of >400 persons. Part of the variety in the size of their social network may be determined by the size of the rural community, but this indicator also signals that many neighbouring farmers belong to the same rural community (as a working unit) and have worked together for agricultural production for several decades.

The OLS regression results in Table 2 indicate which determinants help to explain the abandonment share. The general performance of our abandonment model is satisfactory, with an *R*^2^ of 0.37 (adjusted *R*^2^ = 0.35). The results show that almost all the tested variables are relevant to farmland abandonment. First, the amount of farmland per household member matters: households that have a lot of farmland relative to the number of persons in the household show larger abandonment shares. The fragmentation of farmland—expressed as a low value for the largest parcel index—is also linked to higher abandonment levels. Interestingly, the distance to the furthest parcel is not associated with the amount of abandonment. This suggests that the parcel size and other factors are more important.

**Table 2:** Results for the OLS regression explaining the farmland abandonment share per household (percentage)

Determinant	Coeff.	(Std Err.)
Parcel characteristics		
Farmland area per person	0.04***	(0.01)
Largest parcel index	−0.11***	(0.03)
Distance from the farmhouse to the furthest parcel	0.00	(0.01)
Location characteristics		
Distance from the farmhouse to the main urban districts	0.01	(0.02)
Distance from the farmhouse to the nearest minor road	−0.01*	(0.01)
Distance from the farmhouse to the nearest water	0.01*	(0.01)
Fluvo-aquic soil at the household location	−0.06***	(0.02)
Paddy field	−0.18***	(0.02)
Slope at the farmhouse location	−0.01	(0.01)
Located within a farmland preservation area	−0.06^**^	(0.03)
Farming practices		
Cash crop dominated	0.11***	(0.03)
Vegetable and fruit dominated	0.15***	(0.02)
Ornamental trees	0.01	(0.03)
Mixed crop species	0.05*	(0.03)
Area transferred out	−0.05***	(0.01)
Household characteristics		
On-farm income per person	−0.02***	(0.00)
Adult family members with at least high school education	0.05*	(0.03)
Number of community connections	0.01*	(0.01)
Constant	0.10	(0.14)
*R*^2^	0.37	

Observations = 592. Appendix 2 lists the Pearson correlation coefficients between the included variables

*significant at 10%, **significant at 5%, and ***significant at 1%

Not all the location characteristics assert a large influence on abandonment. Accessibility does not seem to be a strongly differentiating factor in the study area, and only slightly significant effects are found for the distance to the road infrastructure and water. From the range of geographical factors, only the soil type proves to have a significant impact on abandonment. As expected, the presence of the most favourable soil type (fluvo-aquic) helps to limit abandonment. Moreover, paddy fields show less abandonment, possibly indicating the importance of the water supply. The slope at the farm location does not seem to influence the share of abandonment per household, which may be due to the fact that the slopes are fairly gentle across the area. We also tested the model specifications with other geographical characteristics (elevation and rainfall) but did not find these to be relevant either. This again may be attributed to the fact that these conditions are rather uniform and favourable for farming. However, we did find that farmland belonging to a farmland protection area shows lower abandonment levels.

Farming practices are an important determinant of abandonment. Compared with farmland dominated by staple food, farms dominated by cash crops, vegetables, and fruits show higher abandonment rates, while we did not find ornamental trees to have a significant effect. In addition, farms with mixed crop species have higher abandonment levels. Obviously, the households that transfer (rent) more land out are less likely to experience abandonment than the farms that transfer little to no land out.

The socio-economic characteristics of households are also important for explaining abandonment. Not surprisingly, higher on-farm income is linked to lower abandonment levels. Higher education levels, on the other hand, have a positive and significant impact on abandonment. How well households are embedded in the local community also shows a positive but weaker link with abandonment.

## Discussion

Farmland abandonment near cities has hitherto received little research attention in China. However, our survey shows that this is a fairly widespread phenomenon on Wuhan’s urban–rural fringe: on average 12%^1^ of the total farmland area of the sampled households is not managed. This abandonment share is not much smaller than the national level of 13.5% in 2011 and 15% in 2013 (Gan and Yin 2015). In total around 390 ha of farmland are abandoned along the studied transects. If we consider the abandonment share along the selected transects to be representative of farming conditions in the farmland surrounding Wuhan, the total abandoned area could be around 2380 ha (based on the share of the abandoned area accounting for total sampled area 12%). We discuss the most important drivers and possible implications for farmland planning below.

### Parcel Characteristics

In relation to the parcel size, households with large amounts of farmland have larger shares of abandonment. This can be explained by the fact that such farms may not have enough hands available for all the activities required to farm successfully. Labour shortage is especially prominent in peri-urban areas with plenty of alternative employment in the vicinity (Tian et al. 2010). In line with our hypothesis, fragmentation also has a clear link with land abandonment: having many (smaller) parcels coincides with higher abandonment levels, as has also been found in earlier research in Europe and China (Pointereau et al. 2008; Su et al. 2011). Such fragmentation is often the result of dispersed urban development, for example, road infrastructure (Deininger et al. 2012; Su et al. 2014). For land use planning, this indicates that it is important to concentrate urban development in specific regions and leave room for coherent, vital farmland in others. We did not find that parcels located further away from farmhouses are more likely to be abandoned, as was found by, for example, Mottet et al. (2006) and Prishchepov et al. (2013). This most likely relates to the fairly short distance to the nearest parcel in our case study (on average 350 m).

### Location Characteristics

In our analysis accessibility was found to have a limited impact on abandonment. This can be related to the fact that the area is fairly homogeneous and accessibility does not greatly limit the farming conditions. We did not find proof for the negative impacts of urban proximity on the intensity of farmland use that were put forward by Sinclair (1967) or Pointereau et al. (2008). This likely reflects the fact that Chinese farmers do not own their land and do not pay a land rent that is influenced by competing urban uses. Consequently, they are not faced with the high land rents that make investments unattractive in capitalist economies, as was observed by Sinclair. In fact, according to the Land Administration Law of the PRC (PRC 2004), the rural community that owns the land has the right to withdraw farmland from farmers who have not been cultivating their land for a certain number of years (Jin and Deininger 2009). This provides farmers with a clear incentive to keep farming, and they may decide to stop cultivation only when the benefits of alternative options are sufficiently large, the farming conditions are truly unfavourable, or urban development is expected in the near future. The latter may be the case near minor roads, where farms were found to have slightly higher levels of abandonment. This finding differs from studies that emphasized the importance of the road infrastructure for limiting transport costs for farming and allowing easier access to markets (Strijker 2005; Terres et al. 2015). In the urban–rural fringe setting of our case study, the negative impacts of roads on farming conditions seem to prevail. Easily accessible farmland, for example, is more prone to urban development, providing farmers with less incentive to invest and manage these lands (Zhong et al. 2011). In addition, these areas are affected more by human disturbance, as suggested in other studies (e.g. Díaz et al. 2011), and thus require additional protective measures, such as fences to prevent vandalism, trespassing, and theft (Eagle 2009; Heimlich and Anderson 2001).

Most geographical characteristics (slope, elevation, and rainfall) were not found to be relevant to explaining farmland abandonment, again suggesting that these conditions are rather uniform and favourable for farming. The strong negative relation between farmland abandonment and paddy fields implies that water is important for cultivation. Combined with the lower abandonment rate for distance to water, we can conclude that, even in Wuhan, where water is plentiful, the water supply and irrigation opportunities are important. In addition, we found that the local soil quality matters for limiting abandonment. This is in line with earlier findings that identified a lower soil fertility level as one of the main drivers of farmland abandonment (e.g. Rey-Benayas et al. 2007). This is a reminder that urban development should preferably be steered away from the more fertile soils that are important for agriculture. Our study found evidence for the potential benefits of such policies, as farms located within farmland preservation areas were identified as having lower abandonment shares. Protective policies can ensure stable land use rights that favour longer-term investments (Prishchepov et al. 2013).

### Farming Practices

We found that farming practices matter for explaining the observed abandonment shares on the urban–rural fringe, but this relation does not follow a straightforward economic logic. Farms dominated by cash crops or vegetables and fruit on average have larger abandonment shares than staple food-dominated farms, which may be due to the latter one is generally less labour intensive and more land intensive. This is counterintuitive, as the latter type of farming is likely to result in a lower farm income and our result may thus indicate that other motives than optimizing the farm income play a role here. Possible explanations are that efficient cash cropping requires labour that is scarce in this peri-urban area (Bidogeza et al. 2009). In fact, several studies have indicated that off-farm income opportunities result in labour shortages, which may ultimately lead to lower intensities of agricultural land use and farmland abandonment (Chen et al. 2009; Jiang et al. 2013; Liu and Li 2006; Wu et al. 2011). Another explanation is that peri-urban farmers are generally less dependent on the farming income (Heimlich and Barnard 1992, Marsden et al. 1986). In our survey, we found that the average reported farming income per person was generally low and almost equal for the different crop types (ranging from 1450 to 1650 yuan/year). This is low compared with the average income of farming-related activities in Wuhan municipality (31,540 yuan/year) and negligible compared with the average municipal income obtained by employees working in, for example, manufacturing (59,943 yuan/year; see Hubei Provincial Bureau of Statistics 2016). Thus, for most households farming will not be an important income-generating activity, and it is likely that the time and effort that they devote to agricultural practices are limited (Liu et al. 2010). They may, however, decide not to abandon their land completely to retain the right to use it and keep a fall-back option in case their income from other sources is lost (Ma et al. 2015).

In addition, we found that crop diversity is linked to higher abandonment levels, which contrasts studies that have suggested that reliance on different crop types enhances food safety (Lerner and Eakin 2011) and minimizes the risk of agricultural product price fluctuation and the impact of environmental and other stress factors (FAO 2014; Salvatore and Charles 2005). During the interviews, some farmers indicated that although they were gradually less likely to depend on farming income, they preferred to maintain some form of cultivation for diverse reasons such as limiting the cost of purchasing food from the market, food safety with organic food and maintaining rural roots.

Our analysis also confirmed the importance of the option to transfer land to other farmers to keep it in production. This system allows land to be used more efficiently, as it provides farmers who have limited time and resources with the possibility to let other farmers take over their responsibility (Deininger and Jin 2005; Wang et al. 2015) and enlarge the area that they manage, facilitating mechanization and the application of new technologies (Deininger and Jin 2005). This seems to be especially relevant on the urban–rural fringe, where farmers are more likely to find non-farming work opportunities and face labour shortages and land fragmentation (Chen et al. 2008). Therefore, our results confirm that, on the urban–rural fringe, the land use right reforms are also instrumental in preventing further land abandonment.

### Household Characteristics

On-farm income is an obvious incentive for farmers to continue farming. In addition, on the rapidly developing urban–rural fringe in China, farmers are more likely to continue farming when the income is relatively high (De Bon et al. 2010). This indicates that enhancing the farming income can be an effective strategy to keep farmland near cities in production (Meert et al. 2005). Recent experiments with setting up community-based vegetable gardens in rapidly urbanizing parts of the Philippines have shown that they helped participating families to raise their monthly income by about 20% (Holmer et al. 2013). The proximity to resources and consumers allows farmers on the urban–rural fringe a potentially higher farming income thanks to the shorter supply chains, lower transportation costs, and direct marketing options (Lovell 2010; Zasada et al. 2011). Farmers here may even be able to participate in agro-tourism activities (Yang et al. 2010). Alternatively, they can produce high-value-added products, such as organic food, for which the knowledge and processing techniques as well as favourable market conditions are available in and around cities (Beauchesne and Bryant 1999; Cai and Zhang 2000; Zasada 2011). The agriculture-related opportunities of urbanization can thus outweigh the challenges (Wu et al. 2011).

The fact that households with higher education levels are more likely to leave their farmland abandoned indicates that they are more likely to find (better-paying) employment elsewhere. This finding underpins the suggestion of Wang et al. (2016) that relatively less educated farmers tend to specialize in farming in China. We also tested the impact of the share of household members with an off-farm job but did not find significant results. Neither did the average age of the household members or the share of those aged over 60 years yield significant results for explaining the higher abandonment levels, as we expected from the research conducted on farming in Europe by Terres and Nisini (2013) and Van Vliet et al. (2015). This suggests that relatively young households also abandon part of their farmland in our sample, providing another indication that those individuals who are able to work elsewhere probably do so and leave too few hands available for farm work, as was also found by Zhao (1999).

Finally, we find that households that are active in the local community (based on their number of local connections) are less active in farming. This effect is not particularly strong and may indicate that the region has already transformed into a more urban community, where agriculture has become a less important aspect of daily life.

## Conclusion

In our survey we found that a substantial share of farmland on the urban–rural fringe of Wuhan is not used for agricultural production. This farmland abandonment process is not unique to the city of Wuhan but also occurs around other cities in China (Jiang et al. 2013). The limited availability of labour (in relation to the land area of the farms), fragmented parcels, and off-farm income opportunities seem to be the most important drivers of the abandonment of farmland. Especially larger farms specialised in cash crops or vegetables have higher shares of abandoned farmland. Our results furthermore indicate that farmland conservation policies contribute to keeping land in production. Such policies may be used more extensively to concentrate urban development and steer it away from the most suitable areas for farming to limit further abandonment. Further strengthening the land market and removing the remaining barriers for farmers to transfer their land to colleagues can also help in keeping agricultural land in production. This option proved to contribute to limiting abandonment in our sample, in which the shares of transferred-out land were well below the national average. It would be interesting to analyse the degree to which land is transferred out in other peri-urban regions and which factors inhibit its further uptake.

Several additional factors that lie outside the scope of this paper may also be relevant to explaining farmland abandonment. The regulatory process that steers urban and infrastructure development, for example, is likely to influence the conditions for farming, as it appoints the parcels that at some point in time may be taken out of production. Thus, even before land is claimed for such transitions, farmers may decide to limit their investments in farming, but we could not take this into account due to the lack of information. Moreover, the overall market conditions for farming (e.g. demand, supply, and commodity prices) are important determinants of the profitability of farming options. We considered these in the general context of our analysis and did not look for their impact on land abandonment at the surveyed farms. It is clear, however, that the current farms in the region are typically small and unlikely to generate sufficient revenues to compete with the income from off-farm opportunities. Our analysis also did not specifically address the impact of farm subsidies on keeping farmland in production, but during the interviews several farmers indicated that they considered these to be very low and not influential on their farming practices. This is in line with earlier research that indicated that farmland subsidies only offer a marginal addition to the farming income (Gale et al. 2005; Huang et al. 2011). Moreover, psycho-social factors can be influential to determine farmers’ cultivation practices, for example, adoption of technologies. While these are largely excluded from the current research, which can be further stress on in future study.

Preserving farmland use on the urban–rural fringe will not only contribute to securing food production but will also help in enhancing the provision of its environmental and social services (e.g. improving the air quality or providing opportunities for recreation) to the neighbouring urban population. These services have recently gained considerable policy attention, and peri-urban agriculture is now being promoted in the roadmap for rural vitalization published by the Central Committee of the Communist Party of China (State Council 2018). The balancing of the different landscape demands from farmers and urban citizens suggested by this policy document is now being put into practice in, for example, Beijing (Yang et al. 2016). Urban agriculture and the provision of landscape services to the urban population are fairly new concepts in China, however, and they may not yet be important factors for the preservation of farmland use.

## Appendix 1. Transcription of the Survey Questionnaire

During the survey the farmers were asked the following questions:

1. How many parcels of farmland do you have in total in your household?

2. What is the total area of your farmland (hectares)?

3. What is the area of your largest farmland parcel (hectares)?

4. What is the total abandoned area of your farmland (hectares)?

5. How far is it from your residence to the furthest farmland parcel (km)?

6. How much of your farmland is transferred out to other farmers (hectares)?

7. What is the total area devoted to the following (groups of) crops: staple food; cash crops; vegetables and fruits; or ornamental trees (hectares)?

Note: Staple food crops are: rice, wheat, maize, sorghum, potatoes, sweet potatoes, and green beans; cash crops are: cotton, peanuts, rape, sesame, soybeans, and yellow beans.

8. How many family members do you have in your household?

9. Can you indicate the age for each adult family member?

(1) <18; (2) 18–30; (3) 30–40; (4) 40–50; (5) 50–60; (6) ≥60.

10. What is the total farming income in your household?

11. How many persons in your local community are you familiar with through joint social activities such as sharing food, agricultural knowledge, and labour?

12. Can you indicate the employment status for each adult family member?

(1) Full-time farming; (2) mainly farming (more than 50% of working time spent on farmland); (3) mainly in an off-farm job (less than 50% of working time spent on farmland); (4) a full-time off-farm job; (5) unemployed.

13. What is the highest education level that each of your adult family members has completed?

(1) Primary school; (2) middle school; (3) high school; (4) bachelor; (5) master and higher; (6) not educated.

## Appendix 2. Pearson Correlation Coefficients between Farmland Abandonment and Determinants (with Continuous Variables)

**Table:**

	Share of abandoned area per household	Farmland area per person	Largest parcel index	Distance to the furthest parcel	Distance to the main urban districts	Distance to the nearest minor road	Distance to the nearest water	Slope at the farmhouse location	Mixed crop species	Area transferred out	On-farm income per person	Share of education level at least high school (labourers)	Number of community connections
Share of abandoned area per household	1.00												
Farmland area per person	**0.17**	1.00											
Largest parcel index	**−0.18**	**−0.59**	1.00										
Distance from the farmhouse to the furthest parcel	**0.11**	**0.56**	**−0.43**	1.00									
Distance from the farmhouse to the main urban districts	0.03	0.00	0.02	0.03	1.00								
Distance from the farmhouse to the nearest minor road	**−**0.02	**−**0.04	**−**0.03	0.01	**0.14**	1.00							
Distance from the farmhouse to the nearest water	0.07	0.02	**−0.16**	**0.11**	**0.13**	**−**0.02	1.00						
Slope at the farmhouse location	**−**0.03	0.00	**−**0.07	0.06	0.04	**0.21**	0.04	1.00					
Mixed crop species	0.01	**0.13**	**−**0.08	**0.13**	**−**0.07	0.00	**0.10**	0.03	1.00				
Area transferred out	**−**0.06	**0.22**	**−0.12**	**0.16**	**0.09**	**−0.14**	0.00	**−**0.04	**0.13**	1.00			
On-farm income per person	**−0.18**	**0.46**	**−0.31**	**0.24**	0.01	0.06	0.02	0.02	0.07	**0.11**	1.00		
Adult family members with at least high school	0.04	**−**0.00	**−**0.02	0.03	**−**0.02	0.05	0.01	**−**0.01	0.01	**−**0.02	**−**0.00	1.00	
Number of community connections	**−**0.06	**−**0.03	**−**0.04	0.03	**−**0.02	0.06	0.03	**−**0.04	**−**0.03	0.06	0.00	0.13	1.00

Observations = 592. Values in bold are significantly correlated at the 0.05 level

## Appendix 3. Point Biserial Correlation between Farmland Abandonment and Determinants (with Dummy Variables)

**Table:**

	Share of abandoned area per household
Fluvo-aquic soil at the household location	**−0.12**
Paddy field	**−0.42**
Located within a farmland preservation area	**−**0.05
Staple food dominated	**−0.13**
Cash crop dominated	0.07
Vegetable and fruit dominated	**0.11**
Ornamental tree dominated	**−**0.07

Observations = 592. Values in bold are significantly correlated at the 95% level

## Footnotes

1 This is the value averaged by the total farmland area of the sampled households.
